# Population Dynamics of a Salmonella Lytic Phage and Its Host: Implications of the Host Bacterial Growth Rate in Modelling

**DOI:** 10.1371/journal.pone.0102507

**Published:** 2014-07-22

**Authors:** Sílvio B. Santos, Carla Carvalho, Joana Azeredo, Eugénio C. Ferreira

**Affiliations:** Centre of Biological Engineering, Universidade do Minho, Braga, Portugal; University of Erlangen-Nuremberg, Germany

## Abstract

The prevalence and impact of bacteriophages in the ecology of bacterial communities coupled with their ability to control pathogens turn essential to understand and predict the dynamics between phage and bacteria populations. To achieve this knowledge it is essential to develop mathematical models able to explain and simulate the population dynamics of phage and bacteria. We have developed an unstructured mathematical model using delay-differential equations to predict the interactions between a broad-host-range *Salmonella* phage and its pathogenic host. The model takes into consideration the main biological parameters that rule phage-bacteria interactions likewise the adsorption rate, latent period, burst size, bacterial growth rate, and substrate uptake rate, among others. The experimental validation of the model was performed with data from phage-interaction studies in a 5 L bioreactor. The key and innovative aspect of the model was the introduction of variations in the latent period and adsorption rate values that are considered as constants in previous developed models. By modelling the latent period as a normal distribution of values and the adsorption rate as a function of the bacterial growth rate it was possible to accurately predict the behaviour of the phage-bacteria population. The model was shown to predict simulated data with a good agreement with the experimental observations and explains how a lytic phage and its host bacteria are able to coexist.

## Introduction

(Bacterio)phages, or bacterial viruses, can be found wherever bacteria exist which includes every conceivable habitat as a result of their bacterial parasitism. Their presence in the biosphere is especially predominant in coastal water and in the oceans presenting an excess of 10^7^–10^8^ phage particles per millilitre and even higher concentrations in lakes. Phages are also abundant in pelagic marine environments and pointed as responsible for a significant loss of bacterioplankton. Roughly 10^9^ phages per gram can be found in marine sediments and comparably high numbers in other sources like sewage and faeces, soil, sediments, deep thermal vents and in natural bodies of water. Phages are an extremely diversified group and it has been estimated that ten bacteriophage particles exist for each bacterial cell, accounting for an estimated size of the global phage population to be approximately 10^31^ particles, making phages the most abundant living entities on earth [1]–[12]. As a consequence of this high prevalence and ubiquity, even rare phage-induced events will be represented at a high frequency at the global level [7]. This fact, coupled with phage primitiveness and ability to infect bacteria, together with the accumulating data from both phage and bacterial genome sequencing projects, have highlighted their ecological impact acting as agents in the recycling of organic matter (including cells), their key role in the adaptive evolution of bacteria and also as tools in molecular biology and epidemiology [2], [13]–[16]. The existing interactions between phages and bacteria, as well as all the predator–prey dynamics, have long been recognized to play a central role in the structure of ecological communities, or, in this particular case, in the relative proportions of different bacterial species or strains in a community [7], [17].

The increasing problem of antibiotic-resistance bacteria together with the environmental costs caused by the use of chemotherapeutic agents, have motivated the renewed interest in the use of phages as alternative antimicrobial agents in pathogens control [18]–[21]. For an efficient therapeutic use of phages it is also vital to understand the phage-bacteria interactions in order to design efficient protocols, optimize phage production, determine optimal dose concentration and predict the outcome of phage therapy.

In order to understand phage ecology it is imperative to study the interaction between phages and their bacterial hosts making use of (and developing) mathematical models. However, the knowledge of the interactions between virus–host systems in their complex natural environments is still limited [20], [22]. To improve this knowledge it is generally accepted that the development of mathematical and computer simulation models is essential. Such explicit models deal with phages density-dependent replication characteristics and have been used to study the population and evolutionary dynamics of phages [22]–[25]. It is thus critical to first obtain basic information on the behavior of specific virus–host systems during controlled conditions in the laboratory which may disclose some of the peculiar kinetics present even in complex environments, consequently predicting the outcome of an encounter between phage and bacteria [22], [26]. Such studies will enable to fill the existing gap between mathematical models and natural communities by allowing the comparison between the outputs of mathematical models and the experimental data obtained from controlled, reproducible and easily manipulated biological systems, before conclusions can be extrapolated and applied to uncontrolled and complex natural systems [27]. If experimental results do not reflect the simulated ones, then the residues will be used to refine the model. These iterative experimental tests and refinement of the simulations allow for the understanding of relationships otherwise difficult to observe [25].

The first model which attempted to explain the kinetics of phage-bacteria interactions was developed by Levin (1977) from which subsequent models were adapted from [24]. According to the models developed so far, phage-bacteria population dynamics are typically modelled as three interacting populations: susceptible uninfected bacteria, phage-infected bacteria, and free-phage, which depend on phage growth kinetic parameters such as burst size, latent period, and adsorption rate [20], [26].

The work presented herein aims at developing a mathematical model able to predict and explain the basic behaviour of the phage-bacteria population dynamics of lytic phages based on fundamental phage-bacteria biological parameters. Such a model was here developed and the output was found to mimic the experimental data. The key innovative aspects of the model are the incorporation of a normal distribution equation that rules the values of the latent period and the integration of a function that describes the variation of the adsorption rate according to the bacteria growth rate.

## Materials and Methods

### Media

LB broth, Miller (Sigma-Aldrich Inc., St. Louis MO, USA) was prepared according to the manufacturer's instructions. Agar plates were prepared by adding 1.2% of agar (Applichem, Darmstadt, Germany) to the LB broth and Molten Agar was prepared by adding 0.6% of agar to the LB broth. Minimal Medium was prepared based on M9 medium [28] using the following: Na_2_HPO_4_ 6 g/L, KH_2_PO_4_ 3 g/L, NH_4_Cl 1 g/L, NaCl 0.5 g/L, MgSO_4_ 0.12 g/L, and glucose 5 g/L.

### Bacteriophage and Bacteria

The *Salmonella* phage PVP-SE1 isolated from a Regensburg (Germany) wastewater plant as part of the European Project “PhageVet-P”(FP6) was used along with its host, *Salmonella enterica* serovar Enteritidis strain S1400 which belongs to the University of Bristol private collection [29], [30].

### Determination of Phage Titre

The phage titre, expressed as plaque forming units (PFU), was determined using the Plaque Assay Modified with Antibiotic (PAMA) in LB medium as described by Santos *et al.* [31].

### Single-Step Growth Curve Experiments

These experiments were carried out at 37°C, 120 rpm in minimal medium as described by Sambrook and Russell [28], using an overnight pre-inoculum of the bacteria in the same medium. The experiments were made in duplicate and repeated in two different occasions.

### Adsorption Rate

Adsorption rate (*δ*) was determined based on Kropinski [32] using cells grown in minimal medium. Briefly, phage was added to an initial log phase of bacteria suspension at a multiplicity of infection (MOI) below 0.01 to assure that each phage has the opportunity to bind to one, and only one, bacterium. At fixed time intervals, 0.1 ml sample was taken and immediately diluted in 0.89 ml SM buffer (100 mM NaCl, 8 mM MgSO_4_, 50 mM Tris-HCl at pH = 7.5) and 0.01 ml chloroform. Dilution was immediately vortexed and centrifuged at 9000 g for 5 min. Serial dilutions were carried in SM buffer and phage titre determined. The adsorption rate was calculated by the least squares fitting of the data to equation 1 [33].
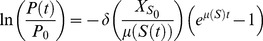
(1)where *μ(S)* is the bacteria multiplication rate, *δ* is the adsorption rate, *X_S_* is the susceptible uninfected bacteria concentration, *P* is the phage concentration and *t* is the time. The subscript 0 refers to concentration at initial time.

This equation was obtained by solving the differential equations 2 (valid for 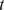 < latent period) and 3, representing the phage and host dynamics respectively:
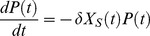
(2)

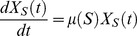
(3)


The bacteria multiplication rate depends on the substrate concentration and is modelled by the Monod kinetics equation 4: 
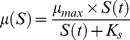
(4)


To determine the maximum rate of exponential growth (*µ_max_*) and the half-saturation constant (*K_s_*), an overnight pre-inoculum of the bacteria in minimal medium was transferred to fresh liquid minimal medium containing different concentrations of glucose (0.01–8 g/L of glucose) in order to obtain an Optical Density (OD) at 600 nm of 0.07 and incubated aerobically at 37°C with 120 rpm agitation. Successive determinations of bacterial cell concentration were accomplished every 20 min by reading the OD at 600 nm. The determination of the two parameters mentioned above was made by the least squares fitting of the data to the Monod's model (equation 4). Glucose (substrate) concentration was measured using the kit Glucose-TR (Spinreact, Spain) according to the manufacturer's instructions. The amount of substrate needed for a new bacterium (*α*) was determined using equation 5.
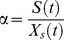
(5)


### Infection Assays

In these assays an overnight pre-inoculum of the bacteria in minimal medium was transferred to fresh liquid minimal medium in order to obtain an OD at 600 nm of 0.07 and incubated aerobically at 37°C with 120 rpm of agitation. Phages were added to the bacterial suspension during initial log phase in order to obtain the desired final concentration. The resultant suspension was incubated aerobically at 37°C with agitation (120 rpm) and samples were collected at fixed intervals to determine bacteria concentration through OD, phage concentration through PAMA and glucose (substrate) concentration using the kit Glucose-TR. These experiments were carried on in a 250 ml Erlenmeyer flask and in a 5 L bioreactor operating in batch mode and using an air inflow of 1 volume/volume of medium.

### Model simulations

The DDEs were solved in Matlab (The Mathworks, Natick, MA, USA) using the *dde23* function/solver. The *fminsearch* function in Matlab was also used for least squares parameter fitting.

To assess the differences between the simulations obtained by the model and the experimental data, we have calculated the root-mean-square deviation (RMSD) normalized by the range of observed values of the simulated variable (NRMSD). Values are presented in percentage. Given the data range of each variable, NRMSD was calculated using the log10 values of the data from total bacteria and free phage to avoid favouring the simulated lower values. In the case of substrate the natural values were used instead of log10. Since the NRMSD is based on the residuals, the closer the value is to zero the best is the simulation fitting to the experimental data. It is important to mention that besides its good accuracy NRMSD cannot be used to compare between variables, but only for a particular variable since it is scale dependent.

A relative NRMSD (r.NRMSD) was calculated when assessing the deviation of the model output to a base-case, corresponding to the quotient of the obtained NRSMD due to the parameter variation and the NRMSD of the base-case. To easily interpret the values, when r.NRMSD is below the unity the value presented corresponds to 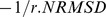. This way, a value of 2 means that the NRMSD is twice higher the NRMSD of the base-case, while a value of -2 means that the NRMSD is twice lower the NRMSD of the base-case and corresponds in this case to a better fitting of the simulation to the experimental data.

## Results

Phage and bacteria interact with each other in a similar way to that of a predator and its prey. The simplest classical model explaining such behaviour is that of Lotka-Volterra. In this simple model the number of preys increase in the absence of predators and decreases as a function of their number. On the other hand, the number of predators decreases in the absence of their substrate (preys) and increases in its presence, proportionally [17], [20], [25].

We have constructed a mathematical model based in Levin *et al.* (1977) [24], a modification of the classical Lotka-Volterra model. The model developed includes the essential characteristics that rule the phage-bacteria behaviour. One of the most important features included is temporal heterogeneity, the existence of a time delay in the conversion of bacteria predation in phage progeny, an intrinsic characteristic of these systems. The proposed model is composed by the following five delay differential equations (DDEs):
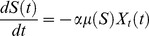
(6)

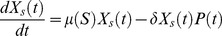
(7)

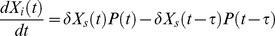
(8)

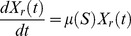
(9)

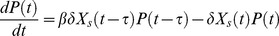
(10)and 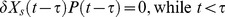
(11)


State variables: *S* is the substrate concentration, *X_s_* is the susceptible uninfected bacteria concentration, *X_i_* is the infected bacteria concentration, *X_r_* is the resistant bacteria concentration, and *P* is the free phage concentration.

Parameters: *μ(S)* is the bacteria multiplication rate, *α* is the substrate needed for a new bacterium, *δ* is the adsorption rate, *τ* is the latent period, and *β* is the burst size.

Total bacteria (*X_t_*) is calculated as:
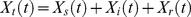
(12)


The substrate *S* is uptake by the susceptible uninfected bacteria *X_s_*, infected *X_i_* and resistant bacteria *X_r_* (i.e. the total bacteria *X_t_*) by an amount of *α* for each newly formed bacterium. The grow rate *µ(S)* of resistant bacteria is assumed to be the same as the sensitive bacteria. The life cycle of the phage starts with its irreversible adsorption to the sensitive bacteria *X_s_* at an infection rate *δ*, proportional to the product of their concentrations given by the law of mass action, and resistant bacteria are obviously not infected by the phage. The phage injects its DNA into the sensitive bacterium (*X_s_*), which becomes infected (*X_i_*), driving the cell to phage replication in order to produce their progeny, and thus it is assumed that the infected bacteria does not grow but still compete for substrate. The phage progeny is not released until the time needed for phage genome injection into the host progeny, phage proteins production and assembly have elapsed, the latent period *τ*, after which the cell lyses releasing an average number of *β* (burst size) new phages able to start a new cycle. This is ensured in the model by using delays in the variables of the differential equations. When 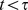, the delayed variables 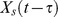 and 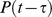 in equations 8 and 10 are set to zero and turning the term 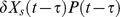 null (equation 11). Consequently, before the latent period, the infected bacteria (equation 8) will only vary as a consequence of phage adsorption to sensitive bacteria, increasing this way the concentration of infected bacteria. On the other hand, free phages will decrease at the same rate and equation 10 will assume the form of equation 2. This behaviour is in accordance with the biological data. As a consequence of the time needed for a bacterium to produce new phages, the population dynamics of a phage-bacteria system does not depend only on the phage and bacteria concentration at time *t* but also on their concentration *τ* hours earlier.

**Figure 1:**
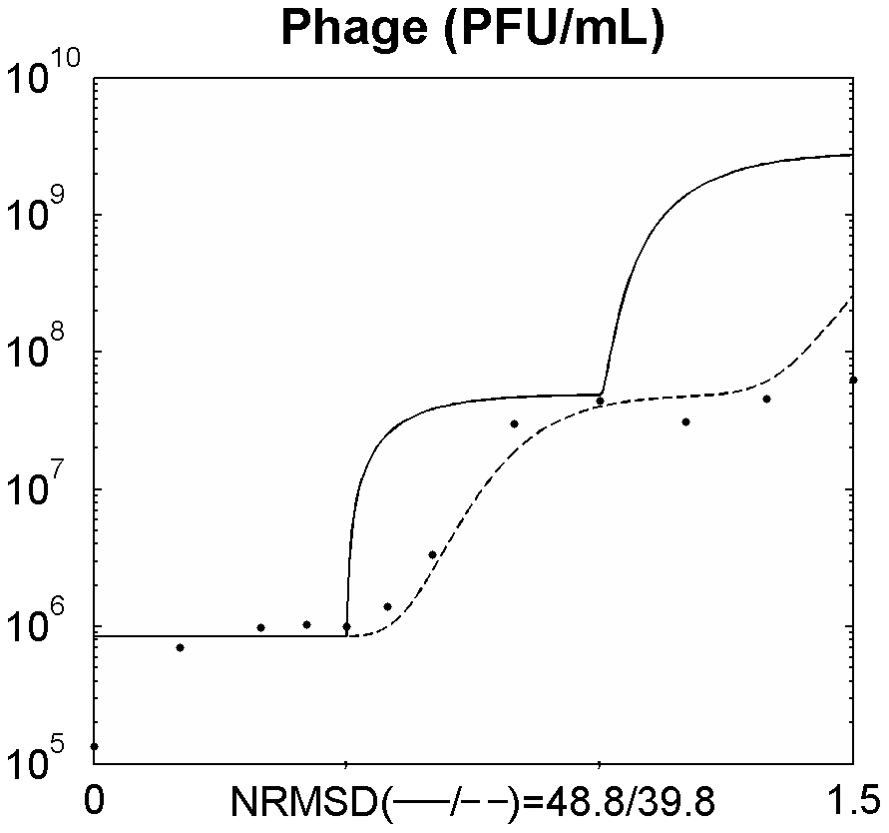
Simulating the one step growth curve using a normal distribution function (equations 6–14). Legend: • experimental data; … data from the model using the average value of the latent period (equations 6–12); ___ data from the model using a distribution of values of the latent period (introduction of equations 13 and 14). The x axis represents time in hours.

The parameters bacterial growth rate, phage adsorption rate, burst size, latent period, and substrate amount needed for a new bacterium, were determined through independent experiments as described in the section “Materials and Methods” and are listed in Table 1. The model was used to simulate the data obtained during the one-step growth experiments and assess the validity of the determined parameters (Figure 1).

**Table 1:** Phage-bacteria interaction parameters determined experimentally.

Parameter	Symbol	Values	Units
Maximum bacterial growth rate	*µ_max_*	0.356	h^−1^
Half-saturation constant	*K_s_*	390	mg L^−1^
Substrate for a new bacterium	*α*	1.23×10^−3^	mg L^−1^
Burst size	*β*	58	PFU CFU^−1^
Phage adsorption rate	*δ_r_*	1.00×10^−9^	ml CFU^−1^ PFU^−1^ h^−1^
Latent period	*τ*	0.5	h
Rise period	*ρ*	0.55	h
lambda parameter (eq. 15)	*λ*	160	
bacterial growth at *δ_r_*	*mS_r_*	0.326	h^−1^

It was observed that using the model as presented above, the data generated showed an overgrowth of the phage population in comparison to the experimental data due to a rapid increase of phage titre right after the latent period. In order to overcome this limitation, a rise period (*ρ*) was introduced in the model to soften the predicted release of the phage by its host. This was accomplished by introducing a normal distribution function (equation 13) with an average 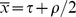 and standard deviation 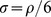.
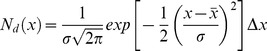
(13)with 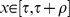 and 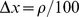.

The normal distribution function when multiplied by 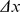, the increment in 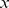, will rule the distribution of the latent period determining the proportion of bacteria that will burst at each time 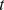 and which were infected at an elapsed time period between 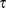 and 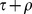 (or 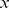). The proportion of bacteria that will burst with a latent period *x* will then be multiplied by the number of bacteria infected *x* hours earlier giving the number of bacteria that will burst with a latent period of *x* in each iteration step (equation 14):
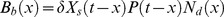
(14)with 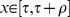.

The sum of all bacteria bursting with a latent period between 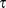 and 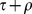, that is 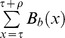, will give the number of bacteria that will burst at each time 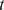, and thus will replace the term 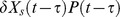 in equations 8 and 10. The introduction of such a function enabled a better agreement between the simulated and the experimental data (Figure 1).

**Figure 2:**
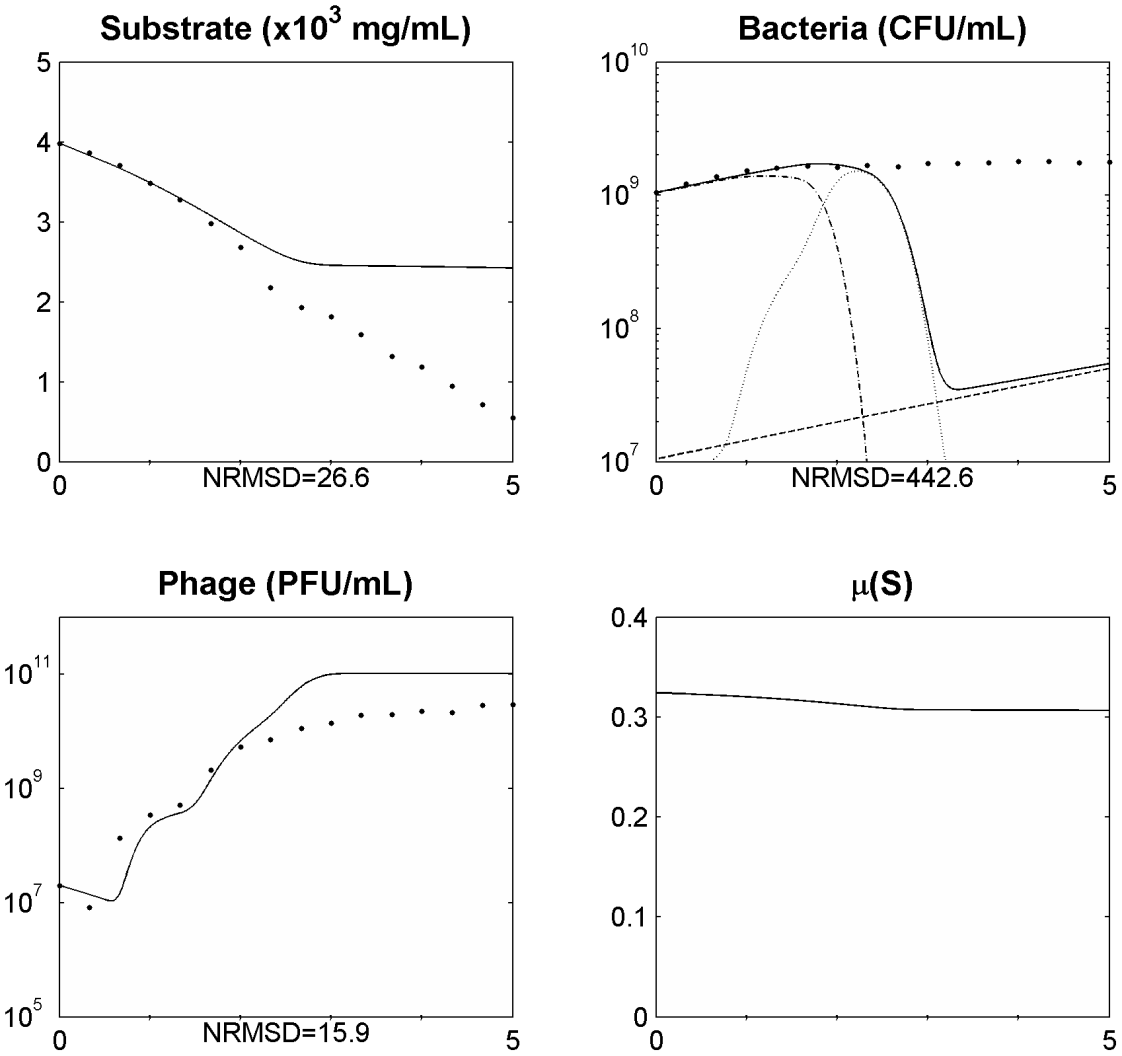
Simulating the phage and bacteria population dynamics using a distribution of values of the latent period (equations 6–14) in a 250 ml Erlenmeyer flask for a MOI = 1.8E10^−2^. Legend: • experimental data; ___ model simulation; - . - model simulation of susceptible uninfected bacteria (*X_s_*); … model simulation of infected bacteria (*X_i_*); _ _ model simulation of resistant bacteria (*X_r_*). The x axis represents time in hours.

**Figure 3:**
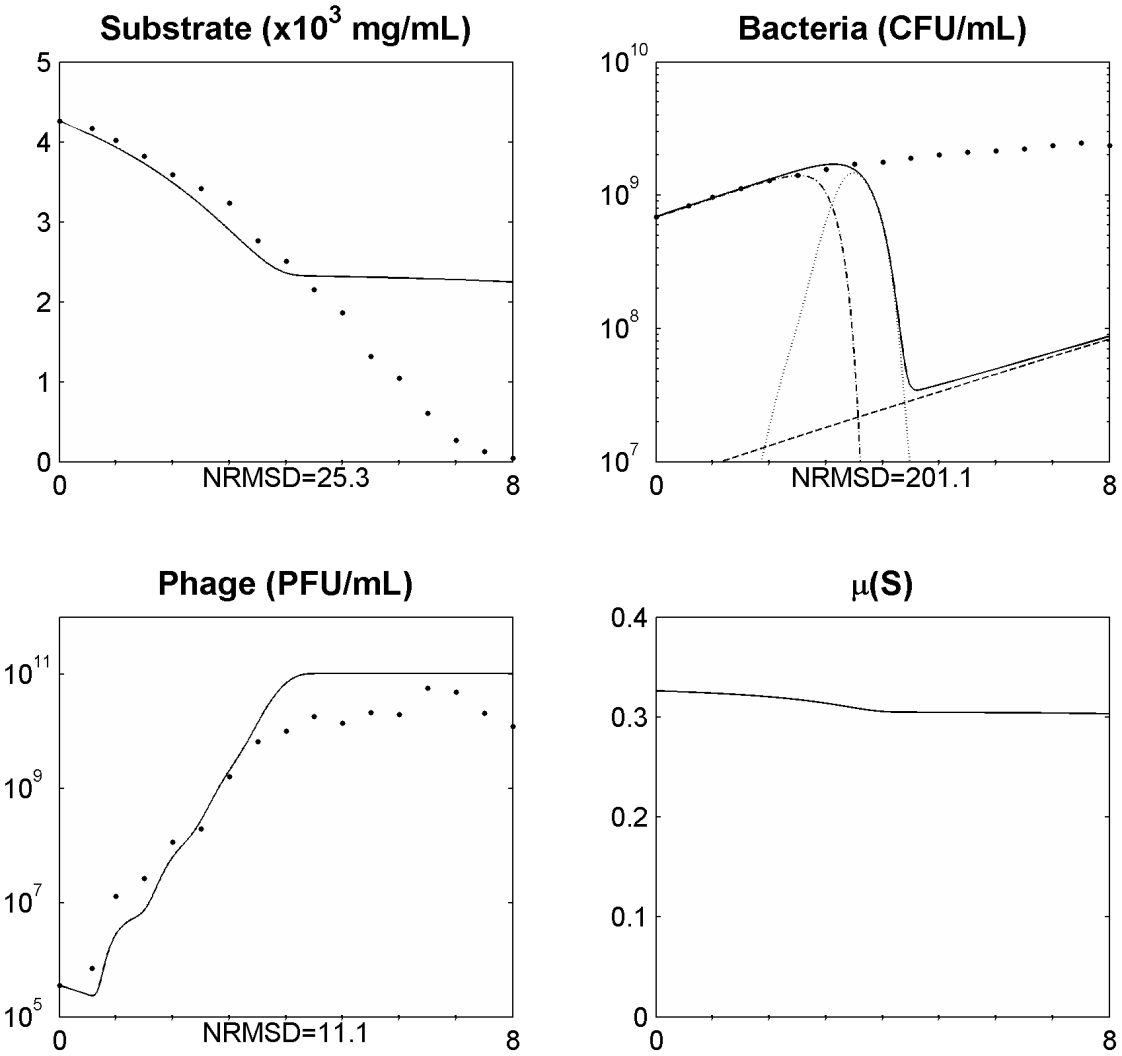
Simulating the phage and bacteria population dynamics using a distribution of values of the latent period (equations 5 and 13) in a 5 L bioreactor for a MOI = 5.1E10^−4^. Legend: • experimental data; ___ model simulation; - . - model simulation of susceptible uninfected bacteria (*X_s_*); … model simulation of infected bacteria (*X_i_*); _ _ model simulation of resistant bacteria (*X_r_*). The x axis represents time in hours.

After assessing the model in a single phage cycle, the model was tested to explain the phage-bacteria behaviour during a longer period. For that, an initial log phase bacterial culture (100 ml) was infected with phage at a MOI of 1.8×10^−2^ in a 250 ml Erlenmeyer flask and the phage, bacteria, and substrate concentrations were determined. This assay was carried out using minimal medium with no addition of substrate during the experiment. This means that the amount of substrate during the experiment will change over time due to bacterial consumption, influencing bacterial physiology. The infection assay was scaled up using a 5 L bioreactor operating in a batch mode with 2 L of medium, where phages were able to infect an initial log phase bacterial culture at a MOI of 5.1×10^−4^. The data obtained was used to assess the suitability of the model presented and the behaviour of the phage-bacterium system at these two scale volumes (Figure 2 and Figure 3).

The data simulated by the model showed a good agreement with the experimental data but only during the first three hours of the experiment. This might suggest that at least one of the parameters in the model may not be constant but instead should vary in the course of the experiment. During the one-step growth experiments it was observed that the time at which the phage was added to the bacterial culture influenced the value of such parameters. In fact, phage infection of stationary grown cultures gives rise to lower burst sizes and higher latent periods than infection of cells growing exponentially (data not shown).

The variation of the burst size was included in the model but no significant improvement was achieved. Since the bacteria size and phage receptors in the cell wall vary with the different growth phases we have thus included a new equation (equation 15) to define the adsorption rate that allows it to vary along the experiment. 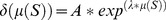
(15)with 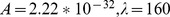


**Figure 4:**
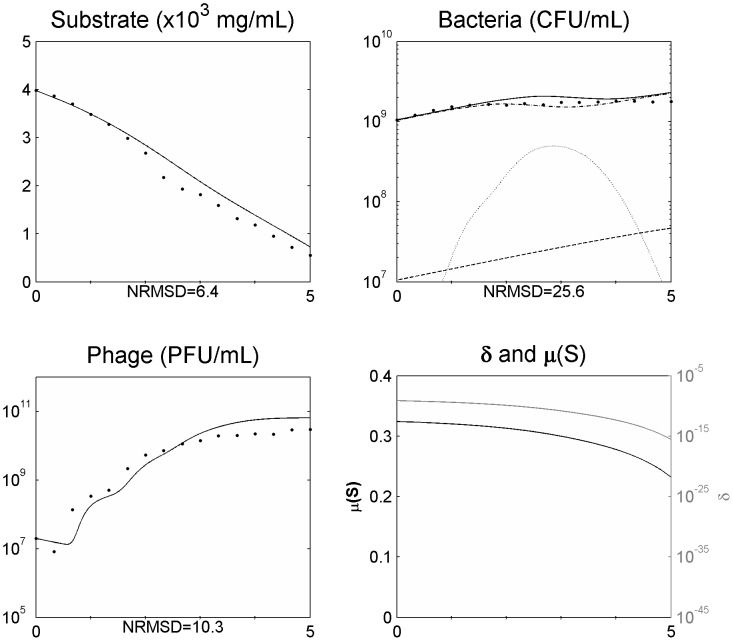
Simulating the phage and bacteria population dynamics using a distribution of values of the latent period and a variation of the adsorption constant (*δ*) as a function of the bacterial growth rate (*µ*) in a 250 ml Erlenmeyer flask for a MOI = 1.8E10^−2^. Legend: • experimental data; ___ model simulation; - . - model simulation of susceptible uninfected bacteria (*X_s_*); … model simulation of infected bacteria (*X_i_*); _ _ model simulation of resistant bacteria (*X_r_*). The x axis represents time in hours.

**Figure 5:**
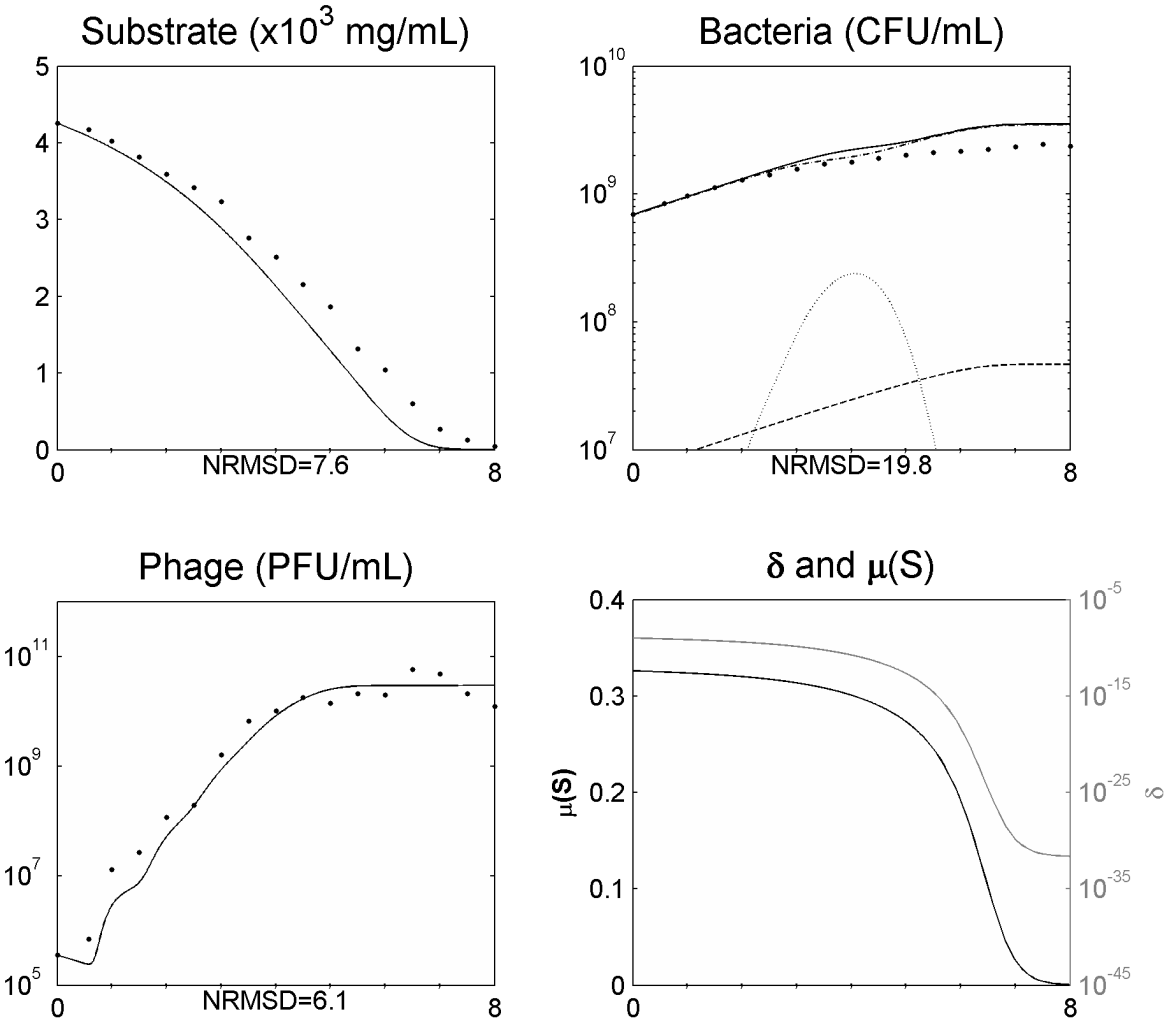
Simulating the phage and bacteria population dynamics using a distribution of values of the latent period and a variation of the adsorption constant (*δ*) as a function of the bacterial growth rate (*µ*) in a 5 l bioreactor for a MOI = 5.1E10^−4^. Legend: • experimental data; ___ model simulation; - . - model simulation of susceptible uninfected bacteria (*X_s_*); … model simulation of infected bacteria (*X_i_*); _ _ model simulation of resistant bacteria (*X_r_*). The x axis represents time in hours.

**Figure 6:**
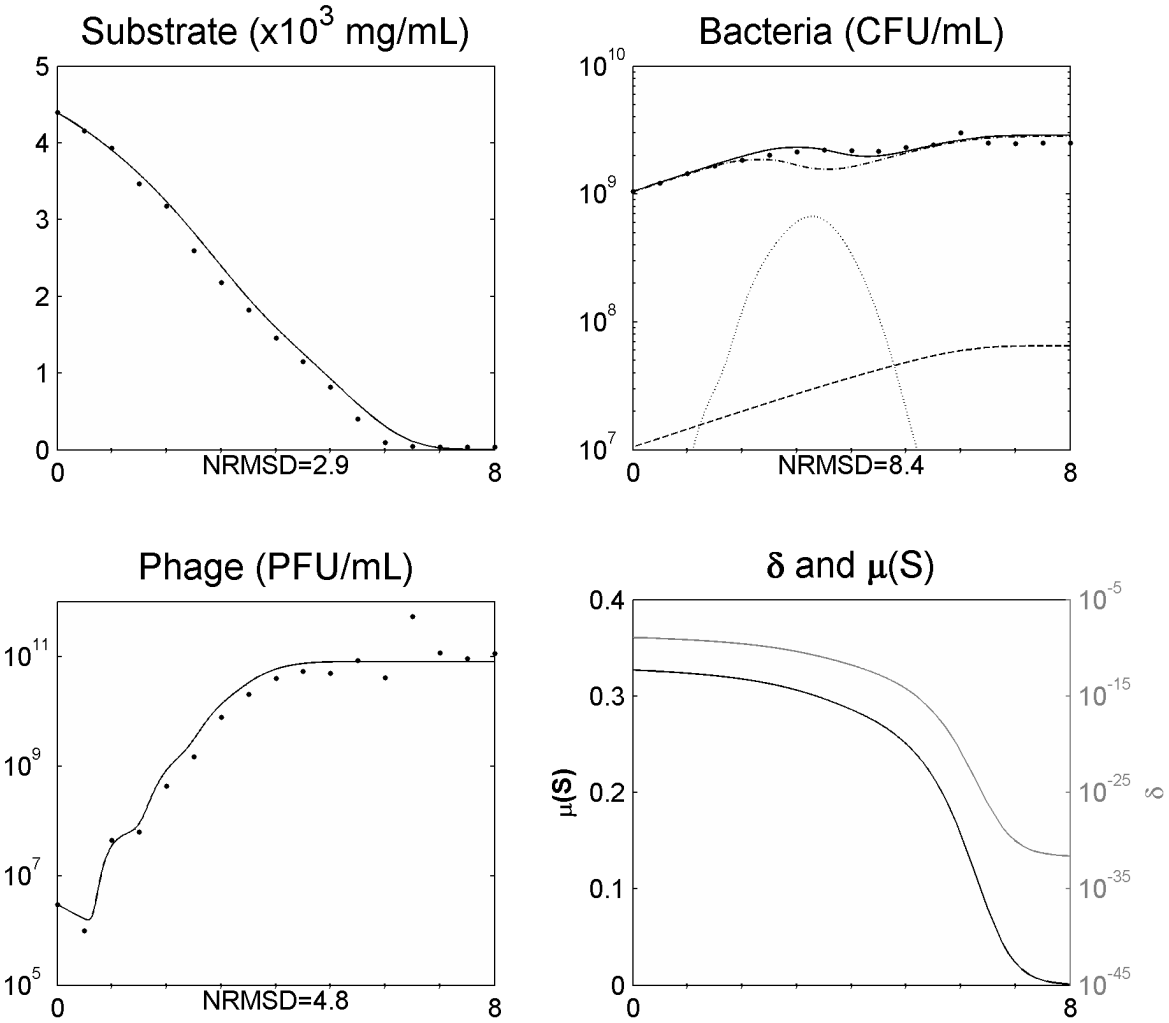
Simulating the phage and bacteria population dynamics using a distribution of values of the latent period and a variation of the adsorption constant (*δ*) as a function of the bacterial growth rate (*µ*) in a 5 l bioreactor for a MOI = 2.8E10^−3^. Legend: • experimental data; ___ model simulation; - . - model simulation of susceptible uninfected bacteria (*X_s_*); … model simulation of infected bacteria (*X_i_*); _ _ model simulation of resistant bacteria (*X_r_*). The x axis represents time in hours.

This equation decreases the phage adsorption rate in function of the bacterial growth rate. The function passes through the experimental determined value of the adsorption rate of 1×10^−9^ for a bacterial growth rate of 0.326 (log phase) and decreases to a value close to zero for a very low bacterial growth rate (stationary cells). Taking into account the experimental coordinates, the parameter *A* was written in order of *λ* which was fitted using the Matlab *fminsearch* function with the experimental data from Figure 2. Since the adsorption is no longer constant, the term 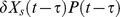 in equation 14 will be replaced by 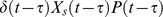. The inclusion of this term in the model allowed a better agreement of the simulated data during all the experiment time periods (Figure 4 and Figure 5). The model was further tested with additional experimental data from an infection assay in the same conditions as the one from Figure 5 but at a MOI of 2.8×10^−3^ (Figure 6). Also in this case, the simulation values presented a good agreement with the experimental data.

**Figure 7:**
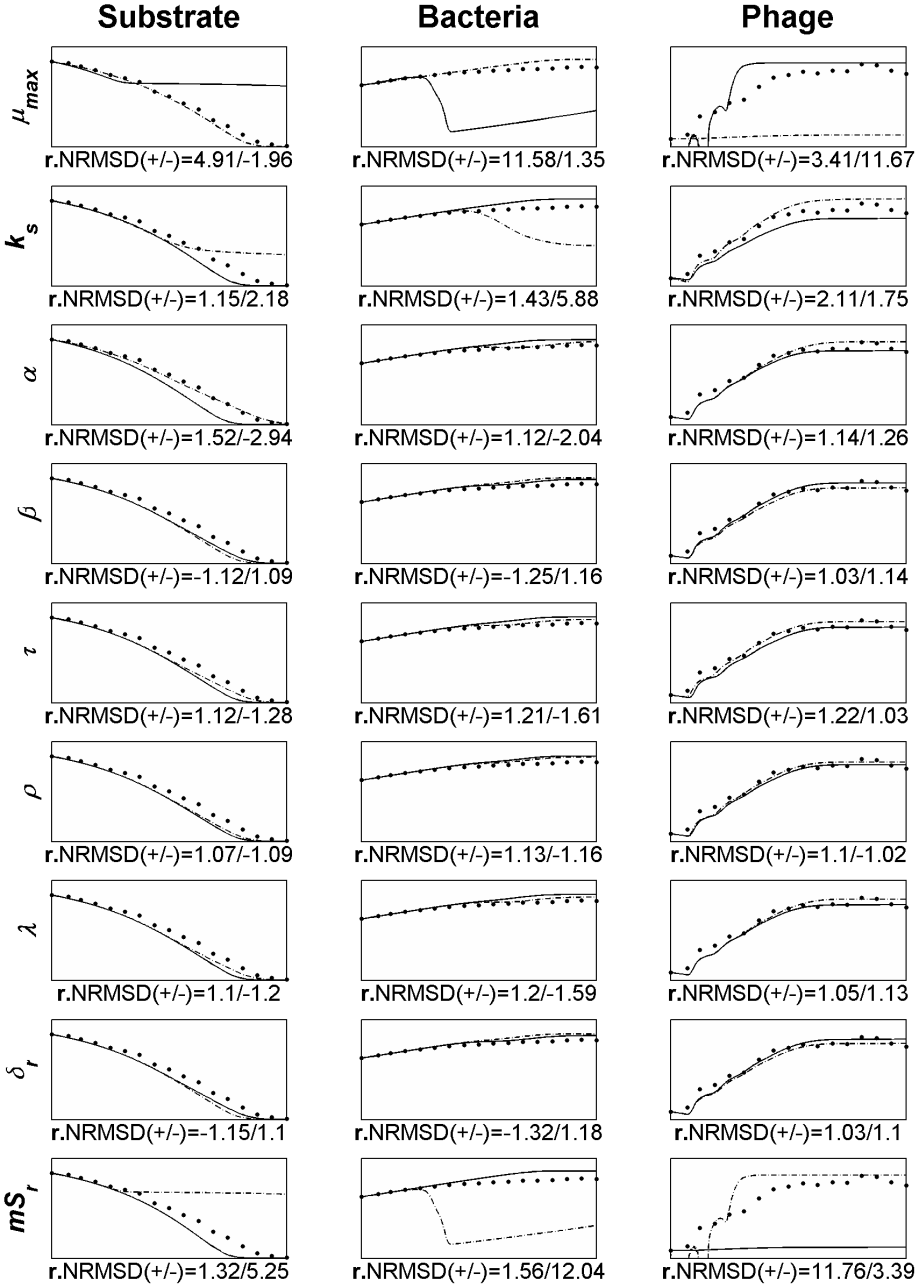
OAT sensitivity analysis of model parameters using as the base-case data from Figure 5. Legend: • experimental data; ___ model simulation when increasing the parameter in 10%; - . - model simulation when decreasing the parameter in 10%. The text on the left side of each line graph identifies the parameter being analysed. *µ_max_*: maximum rate of exponential growth, *K_s_*: half-saturation constant, *α*: substrate needed for a new bacterium, *β*: burst size, *τ*: the latent period, *ρ*: rise period, *λ*: lambda parameter used in equation 15, *δ_r_*: experimental determined adsorption rate, *mS_r_*: bacterial growth rate at which the adsorption rate was calculated, The x- and y-axis are the same as Figure 5 (x: time [1 8] hours, y_Substrate_: [0 5000]; y_Bacteria_: [10^7^ 10^10^]; y_Phage_: [10^5^ 10^12^]).

In order to evaluate the influence of the different parameters (inputs) on the model variables (outputs), a sensitivity analysis was conducted using the OAT (one-at-a-time) method, that is, changing the value of each parameter one at a time for a magnitude of 20% (±10%). This local sensitivity analysis was carried using as the base-case the simulation and data from Figure 5 and all the parameters included in the model were subjected to analysis (Figure 7).

For the sensitivity analysis we have determined a relative NRMSD (r.NRMSD) for each parameter variation which includes in its calculation the value of NRMSD of the base-case (as described in the section “Materials and Methods”). The r.NRMSD enables the determination of the parameter that causes the greatest deviation to the base-case and consequently the parameter for which the model is more sensitive.

**Figure 8:**
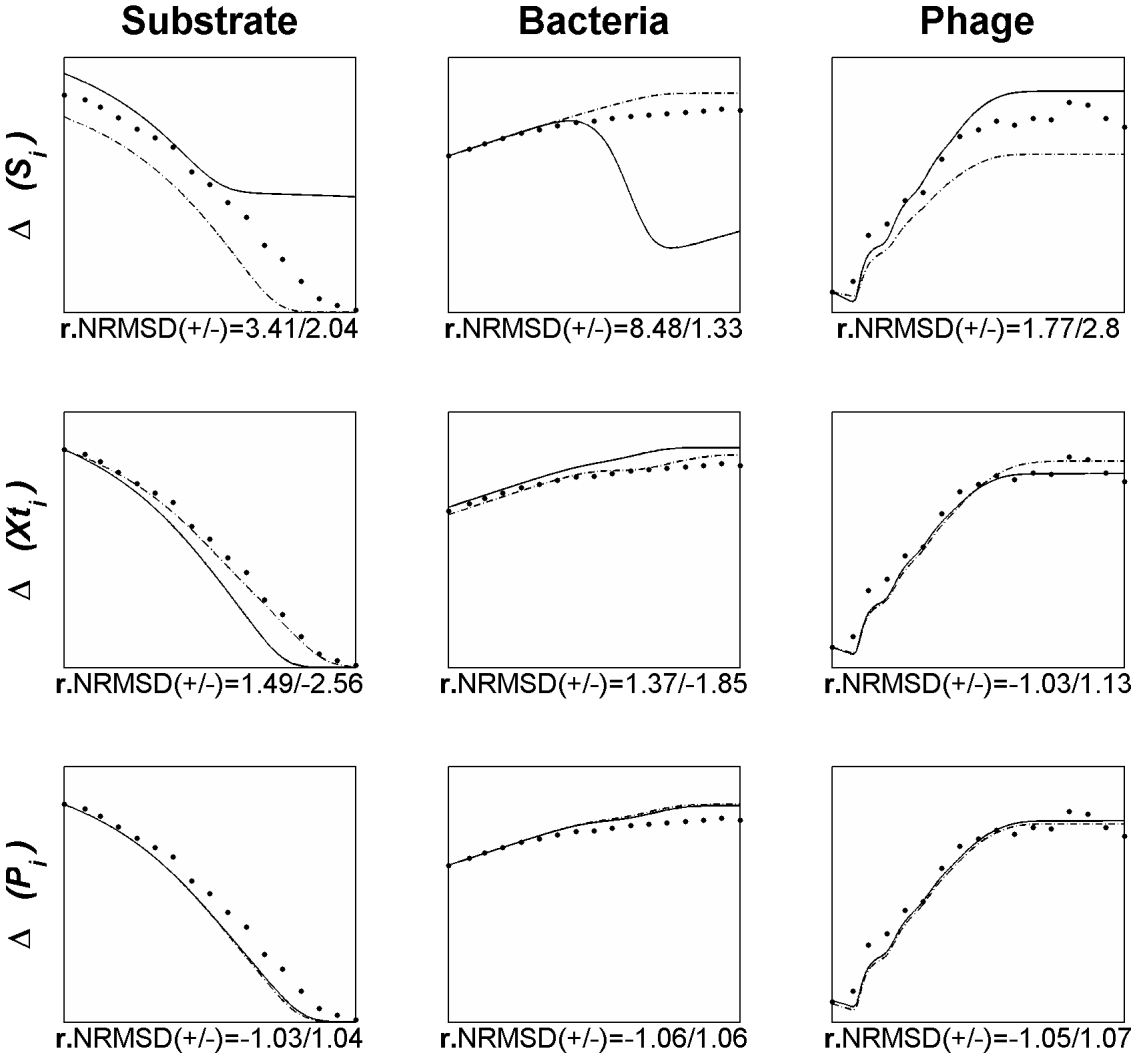
Variations in the model simulation when varying each of the state variables alone in 10% (using as the base case the data from Figure 5). Legend: • experimental data; ___ model simulation when increasing the variable in 10%; - . - model simulation when decreasing the variable in 10%. The text on the left side of each line graph identifies the variable being analysed. Δ*(S_i_)*: variation in initial substrate concentration, Δ*(Xt_i_)*: variation in initial total bacteria concentration, Δ*(P_i_)*: variation in initial free phage concentration. The x- and y-axis are the same as Figure 5 (x: time [1 8] hours, y_Substrate_: [0 5000]; y_Bacteria_: [10^7^ 10^10^]; y_Phage_: [10^5^ 10^12^]).

To assess the outcome of a variation in the state variables we have modelled the outcome of the *Salmonella* phage and its host by changing each of the state variables alone in ±10% (Figure 8), using as the base-case the same data from Figure 5.

## Discussion

### Modelling the phage–bacteria interactions

The unpredictability of the population dynamics of the phage–bacteria interactions is related to the intrinsic self-replicating nature of phages. Therefore, the awareness and understanding of kinetics and dynamics of phages and bacteria is important to predict the outcome of the interaction between these two populations in a given environment. To accomplish this, it is crucial to develop mathematical models able to explain and reveal the interaction between phages and bacteria [25]. When these models produce simulations with a good agreement with experimental data they can be powerful tools for the optimization of phage infection parameters towards the improvement of phage production or for the assessment of phage therapy outcome. Nevertheless, it is important to be aware that it is not possible to include all factors accounting for the population and evolutionary dynamics of the interactions between bacteria and phage in a mathematical model [20].

Structured models (models which consider the behaviour of intracellular metabolic pathways in response to environmental changes) provide a more comprehensive description of microbial behaviour than unstructured models, however they are limited, complex and the majority of reported models are not validated with experimental data, in part due to difficulties for the knowledge *in vivo* of the reaction rates of the implied enzymes [34]. On the other hand, unstructured models describe the production of biomass being more simple but equally useful and descriptive of the experimental reality. Essentially, unstructured models of viruses keep track of virus as well of uninfected and infected host populations [35]. The results generated by such unstructured models can be useful in interpreting large data sets and identifying useful new experiments to perform [36].

### Populations present in the model

We have here developed an unstructured model useful to understand the generic kinetic phenomena of the self-replicating nature of phages. Models describing phage-bacteria population dynamics are characterized by the interaction of three different populations: susceptible uninfected bacteria, phage-infected bacteria, and free phage [37]. Variations in these populations represent the core of a phage-bacteria unstructured model [35]. Another important population considered in the model developed herein consists of resistant bacteria. Even though this population do not interact with phages and generally do not contribute to the other two, its prevalence in a given environment will vary as a result of the interaction between phages and sensitive bacteria. Moreover, in a scenario where resistant bacteria are able to overcome the susceptible bacteria they will obviously compete for substrate with up to the same fitness. Nevertheless, due to the low proportion of resistant cells in the bacterial population and the time length of the experiments, resistant cells did not produce a significant impact in the results. This may constitute a particular case of the phage used in the experimental assays since it presents a broad lytic spectrum (a characteristic found more frequently in *Myoviridae* phages than in other *Caudovirales*) and thus bacteria may have more difficulty in developing resistance against this phage [7], [29], [38].

### Assumptions of the model

Some assumptions were made in the development of the model. The suitability of the model is only for lytic phages and not for temperate phages which can follow the lysogenic cycle not leading to the death of the bacterial cell and thus not acting always as a predator [39]. Phages encounter bacteria at random and will only adsorb to susceptible bacteria which can be infected by only one phage, since infection of the bacterium by a phage can inactivate the remaining receptors [23]. Consequently, the super-infection phenomenon that may exist when the multiplicity of infection (MOI) is high was not included. The infected bacteria do not grow by cell division, a commonly and more accurate assumption in phage-bacteria models, since after infection, phages take control of the bacterial machinery driving the cell only for phage production [23], [37], [40].

Resistant bacteria are assumed to not present a fitness disadvantage over sensitive bacteria and thus their grow rate *µ(S)* is assumed to be the same as the latter [18]. As infected bacterial cells are still metabolically active and requires a significant commitment of host resources in producing phage progeny, it was assumed that infected bacteria compete for substrate with the resistant and susceptible bacteria, an assumption found in other models like the one from Beretta and Kuang [41]. This assumption is corroborated by the work of Middelboe that showed that bacteria continue to uptake thymidine after cell infection until cell lysis, that is, during the lytic cycle of the phage [22]. Also, different studies found that viral production may even increase the rate of glycolysis of infected cells [42]–[45]. The work from Jain and Srivastava compared the major pathways in the infected cell metabolome with those in the uninfected cell. Even though that host metabolism is shifted for phage production diverting resources away from the TCA cycle and from the synthesis of cell wall components in order to upregulate the pentose phosphate pathway, the glucose uptake rates of uninfected and infected cells were found to be similar [36]. Conversely, the model developed by Jain and Srivastava to describe the interaction between the MS2 phage and *E. coli* predicted that the host cell do not grow since the value of the flux corresponding to the biomass production was zero [46]. Moreover, modelling infected bacteria as a growing population increases the phage growth rate, resulting in an increased distance to the observed biological data [37], fact that we also observed.

Acquired resistance of bacterial cells during the experiment time period and the possibility of bacteria to use cell contents of lysed cells as substrate were not included in this model since they are not relevant during the time scale used in this study [18], [22].

### Setting the latent period and inclusion of asynchronous bursts

The factors known to primarily rule phage-bacteria population dynamics are bacteria multiplication rate and substrate consumption, phage adsorption rate, burst size and latent period and thus these factors constitute the model parameters. The distribution of variation in these parameters also plays a critical role [20]. This last parameter, latent period, is an intrinsic characteristic of the phage-bacteria system (within a given environment) and has been neglected in the model presented by Levin and Bull (1996) and treated by Payne and Jansen (2001) as a lysis rate [47], [48]. Without this parameter, simulated data will result in a higher phage growth due to an early release of phage progeny, leading to the appearance of new phages right after phage infection and before the latent period has passed. In the model presented herein, delay differential equations were used to include this temporal heterogeneity by treating the latent period as it is, a time period without phage release.

Experimental determination of the latent period is made through the single-step growth curve and corresponds to the time elapsed between infection and the time at which the phage concentration starts to rise. After this time point, the phage concentration continues to rise during a time period (the rise period) until a plateau is reached, showing that the infected bacteria do not all burst at the same time. These asynchronous bursts are due, in a lesser extent, to the different time at which phages adsorb (which depends on the adsorption rate that is usually very fast) but mainly to different latent periods within the infected-bacteria population, fact that will be reflected in the length of the rise period (*ρ*). Observing the single-step growth curve of a phage (as the one from Figure 1), the higher slope of the curve during the rise period, usually at its midpoint, reflects the majority of the infected cells bursting and releasing the progeny phages. The non-inclusion of these asynchronous bursts in the model is common practice in modelling phage-bacteria populations but it will produce a faster increase in the phage release right after the latent period, enabling phages to start a new cycle much sooner, with the consequent overgrowth of simulated phage concentration when compared to the experimental data and leading to a larger decrease of the sensitive bacteria when compared with the real data [37]. Such behaviour was also observed in our model (Figure 1) as a result of the non-inclusion, in a first approach, of these asynchronous bursts. Figure 1 shows an overgrowth - more than two logs in only one phage cycle - of the simulated phage concentration in comparison to the experimental data. The model was readjusted to deal with the asynchronous bursts by including a normal distribution function (equation 13). The centre of the distribution curve is *τ+ρ/2*, the midpoint of the rise period, which corresponds to the higher number of infected bacteria bursting. Using *ρ/*6 as the value of *σ* enables to include the limits of the distribution curve inside the rise period time length. The inclusion of this normal distribution function will rule the latent period distribution along the rise period by determining the proportion of infected bacteria that will burst at each possible time inside the limits *τ* and *τ+ρ* during the experiment. In contrast to what was observed by Weld and colleagues [37], this modification to the model enabled a much better agreement between the simulated and experimental data, as can be seen in Figure 1. Weld and colleagues distributed the burst over 10 min around the end of the latent period, apparently in the same way as Schrag and Mittler [49], as an even distribution over that period and not as a normal distribution, which seems to be the reason for this divergence.

### Non-eradication of the bacterial population

Understanding the predator nature of phages and their ability to replicate each time a bacterium is infected lead us to speculate that the interaction between these two populations will result in a rapid growth of the phage population and the extinction of all sensitive bacterial population. Indeed, using the model with constant parameters and allowing for a normal distribution of the latent period to simulate the population and evolutionary dynamics of the phage–bacteria interactions, this behaviour is observed (Figure 2 and Figure 3). This has also been reported in other models [17], [20], [24], [47], [49]. Nevertheless, experimental data reported herein and described by several other authors [22], [49], [50] shows that the phage concentration rapidly increases but the sensitive bacteria are not eradicated and are still found in the reactor even after the phage concentration have stopped increasing (Figure 2 and Figure 3). Two reasons have been pointed out to the non-eradication of the sensitive bacteria: the presence of surfaces, biofilms or others that may act as physical shelters to bacteria, consequently preventing phage adsorption [20], [49]; and the physiological state of bacteria, since that stationary cells (due to its reduced metabolism) may preclude phage infection and replication [7], [22]. This means that the bacterial cell needs to be accessible to the phage and in an appropriate physiological state to produce phage progeny [7]. As in the reactor experiment reported herein, the majority of bacteria are suspended and most likely not enclosed in physical shelters, the not eradication of sensitive bacteria is probably due to cells that are not in an appropriate physiological state to produce phage progeny. Indeed, we have observed that as the bacteria growth rate decreases, the rate of phage production also decreases (Figure 2 and Figure 3).

### Physiological changes of stationary cells

The physiology of the bacterial cell will change through the different growth phases. When entering into the stationary phase, which is usually due to the depletion of available main nutritive components, the cell metabolic activity is greatly reduced, the bacterial growth declines due to reduced cell division, the outer membrane presents high degree of local charge heterogeneity, the receptors in the cell membrane may change in quantity and morphology, the bacterial motility is reduced and cells become smaller, in some cases changing their morphology from rod-shaped to cocci [51]–[53]. These changes on the physiology of the bacteria will induce variations in the parameters that rule the interaction between the phage and bacteria. Phage production and cell lysis were found to present a positive correlation with the bacterial growth rate of hosts in bioreactors suggesting a strong dependence of phage-bacteria population dynamics on the physiological state of bacteria [22].

### Dependence of the adsorption rate on the cells physiology

The first step in the growth cycle of a phage is its adsorption to specific receptors on the susceptible bacterial surface representing the fitness of the phage in capturing its host. This characteristic has been found to vary to a great extent with the physiological state of the host cell [20], [23], [54]. An increase in the cell surface, a large number and density of receptors in the host cell surface and a higher motility of bacteria will be reflected in the adsorption rate constant increasing its value [17], [54]. The collisions between the phage and bacteria happen at random Brownian motion and the adsorption rate is dependent on the concentration of both intervenients being typically modelled by the law of mass action [23]. The experimental determined adsorption rate constant will thus represent the density of phage receptors in the host cell surface, the diffusion rate in the medium, the size of both phage and bacteria and the efficiency of phage infection in relation to collisions [55]. Therefore, the adsorption rate constant will strongly depend on the physiological state of the bacterial cells which will in turn produce variations in that parameter during the time period of the experiment. This fact will also produce not only variations in the determined value of the constant between experiments carried at different physiological states of bacteria, but also difficulties in its determination since even slight physiological variations in the host cells may occur during an experiment [54].

### Modelling phage-bacterial populations as a function of the growth rate

Although bacterial cells in stationary phase do not produce, or present minimal production of phage progeny, the resumption of cell growth by addition of nutrients results in an immediate lysis of host cells and phage production [7], [22]. This fact suggests that phage production yield, and consequently the kinetic parameters, do not depend on the cell density or quorum sensing but rather on bacterial growth rate which can be determined by the amount of substrate available following a kinetic model such as that described in Monod's equation (equation 4). Consequently, the phage population (predators) starts to be primarily limited by the number of available preys but when the bacterial growth rate decreases it will be primarily limited by the host physiology (in this case as a consequence of resource availability). Hurley and colleagues [56] also found that the parameters that significantly influenced the simulation outcomes of their *in vivo* model for the SP6 phage-*Salmonella* system were phage adsorption rate and bacterial growth rate, however these authors did not related these two parameters and assumed a constant value in each simulation. Levin and Bull also concluded that a variation in phage adsorption and burst size values in time would eliminate the inconsistencies between the experimental results and their model, which predicted that the phage (combined with the immune defences) should eradicate the bacteria [47]. Even so, those variations were not implemented to avoid complexity of the model. Moreover, Hadas and colleagues observed that the rate of the irreversible step in T4 phage adsorption increased with the host growth rate [57].

Along these lines, we have modelled the adsorption rate as a function of the bacterial growth rate. Thus, phage adsorption will present its maximum when the growth rate equals the maximum bacterial growth rate and will decrease as the bacterial growth rate decreases until reaches a value of zero. This variation in the adsorption rate enabled a good fitting of the simulations with the experimental data for phage, bacteria and available substrate and was able to explain why (and how) sensitive uninfected bacteria are able to coexist with lytic phages (Figure 4 and Figure 5) as has been observed not only in therapy but also in environmental and ecological niches [17], [24], [49].

Like other authors we have also found dependence of the latent period and burst size on the host growth rate. The burst size seems to present a direct variation with that of the growth rate while the latent period presents an opposite variation, that is, the latent period is lengthened and the burst size reduced when the growth rate decreases, reducing the growth rate of phage progeny. Although the burst size presents a wide variation reaching the value of 1 when cells achieve stationary phase, the variation of the latent period is at a lesser extent. The variation of these two parameters as a response to growth rate was already observed [7], [22], [37], [58]. The value of 1 for burst size of stationary cells reinforces the conclusion that infected stationary cells release progeny phage only after resumption of cell growth, which in this case, happens only after plating the cells, since no substrate was added during the experiments. These variations have been introduced in the model but no significant improvement in the simulations was observed. The wide variation of the burst size may be a result of a low adsorption of phage to cells that was already taken into account in the model, which may explain why these variations did not significantly improved the model simulations. Consequently, and due to the increasing complexity that it produces, we did not maintained these modifications for the sake of simplicity.

The dependence of phage fitness, represented by its biological parameters, on the bacterial growth rate will certainly have implications in environmental simulations and in phage therapy. *In vitro* (or in laboratory controlled conditions) studies of the population dynamics of phage and bacteria are usually carried at optimal conditions of bacterial growth which do not happen in the bacteria natural environments or organisms subjected to phage therapy. Therefore, kinetic parameters determined *in vitro* using exponential growth cells in optimal physiological state for phage infection cannot be used straightforward to model phage bacteria interactions in real environments since they will change while bacteria grow, due to variations in the cells physiological state.

Accordingly, the presence of a phage and a sensitive bacterium is not sufficient for infection to occur.

### Sensitivity analysis

The complexity of the model and the number of parameters included turns difficult to understand the existing relationships between the inputs (parameters) and the outputs. Having this in mind we have carried a sensitivity analysis using the OAT method. This enables to vary a parameter and observe its effect on the model output, unambiguously showing the contribution of that particular parameter on the simulation. From this analysis (Figure 7), using the data from Figure 5 as the base-case, we have observed that the parameters that produce a wider deviation from the base case are the bacterial growth rate (*mS_r_*) at which the adsorption rate was calculated (*δ_r_*), the maximum rate of exponential growth (*µ_max_*) and the half-saturation constant (Ks). The analysis showed that the Monod's parameters, and specially its output (bacterial growth rate), exert major influence in the model output. All these three parameters are directly related with the bacterial growth and confirm the influence that it has on the model. As a result, the identified parameters should be the focus of attention and need to be reliably determined. More important than determining the adsorption rate of reference (the one determined experimentally to define the adsorption equation, equation 15) is assessing the growth rate at which it was determined. Furthermore, the influence of the bacterial growth rate, and consequently the phage adsorption to the bacteria, is even higher than the burst size, a parameter commonly used to select for phage fitness. Using the values of the r.NRMSD (the higher absolute value from the ±10% variation) the parameters can be ranked in order of its higher impact on the model output: bacterial growth rate at which the adsorption rate was calculated (*mS_r_*), the maximum rate of exponential growth (*µ_max_*), the half-saturation constant (Ks) and at a lesser extent the substrate needed for a new bacterium (α), the latent period (τ), the lambda parameter used in equation 15 (*λ*), the substrate needed for a new bacterium (*α*), the experimental determined adsorption rate (*δ_r_*), the burst size (*β*) and the rise period (*ρ*).

### Variations in the state variables

Phage, bacterium and substrate concentrations are key variables when setting up conditions to produce phage, to use phages as antimicrobial agents or to understand changes in a phage-bacterium community. To assess the outcome of a variation in these state variables and consequently its influence in a phage-bacterium population we have modelled the outcome of the *Salmonella* phage and its host by changing each of the state variables alone in ±10% (Figure 8). The determination of the r.NRMSD was made using as the base-case the same data from Figure 5):

- Substrate (Figure 8, line 1) – by increasing the initial substrate concentration in only 10% the sensitive bacteria are almost completely eradicated decreasing the total bacteria population (composed mostly by infected and resistant bacteria). The final phage concentration in the other hand is increased. At a first glance the increase in the substrate should favour bacterial growth but this will also increase the number of bacteria susceptible to the phage with a higher growth rate. As stated before, according to our model, the increased bacteria growth rate will increase phage infectiveness through a higher adsorption rate and therefore an equally increased phage progeny. The decrease in the substrate concentration does not have a great impact when comparing with the base-case. There is a slight increase in the bacteria population that will stop their growth sooner due to the depletion of substrate. Also, a slight increase in the phage concentration is observed.
- Bacteria (Figure 8, line 2) – increasing the total bacteria concentration will increase the number of sensitive bacterium able to be infected by phages and consequently it would be expectable an increase in the final phage concentration. Even though, the final concentration of phage decreased. Such behaviour is explained by the decrease in the substrate concentration in the medium due to the increase in bacterial population. This decrease in substrate concentration will lower the growth rate of the bacteria (calculated through the Monod equation, equation 4) that in turn will decline the adsorption rate leading to less phage infections and consequently less phage progeny. Moreover, the growth rate for which the phage adsorption is close to zero will be achieved sooner and thus the phage concentration will also stagnate sooner. When decreasing the total bacteria, beside the lower number of susceptible bacteria, the final phage concentration achieved will be higher than the positive variation of this state variable. This is a consequence of the higher availability of substrate per bacterium, since the substrate was maintained at its base line, which will lead to a higher growth rate of bacteria and in turn a higher phage infectiveness, as explained above. The phage concentration is primarily limited by the lower number of susceptible bacteria (until half the time) but then, the higher growth rate of cells leads to a higher phage population.
- Phage (Figure 8, line 3) - the variation of ±10% in the initial phage concentration produced a very small variation in the final phage and bacteria concentration when compared with the base case. These changes are almost imperceptible showing that small differences (at least up to 10%) in the initial phage concentration will not compromise/improve phage production and phage effectiveness in the control of the bacterial host;

This analysis showed that variations in the initial conditions of the state variables are not those that would be the most expectable. Increase in the bacterial substrate will lead to bacteria reduction and phage increase while decrease in the substrate may enable bacteria survival when confronted with a phage attack and an initial higher population of bacteria, if not supplemented with a higher amount of substrate, will lead to lower phage production. Only the increase of phage titre seems to have the expectable output, a higher final phage concentration and a lower bacterial population, but even so, for a population that reproduces exponentially, the variation is almost imperceptible.

From these variations it is possible to observe that the initial substrate concentration was the state variable that produced the most pronounced changes. From a clinical point of view or for phage production purposes it seems that, in contrary to what would be expected, to reduce the bacterial population or increase the final phage concentration it is advisable to increase the substrate available for the bacteria. Consequently, changes in the substrate may have dramatic impact in bacteria control with consequences for the success of therapy and phage production.

The proposed model clearly shows the major role of the physiological state of the bacteria in the population dynamics of a lytic phage and its respective host. The model contributed to give further insights about the role of the growth rate (as well the parameters that mostly can influence it, as is the case of the maximum rate of exponential growth - *µ_max_* - and the half-saturation constant - *K_s_* - and the state variable initial resource concentration - *S_i_*) in the course and outcome of the interaction between a lytic phages and its respective host.

## Conclusions

In conclusion, an unstructured mathematical model has been developed using delay differential equations to predict and explain the basic behaviour of the phage-bacteria population dynamics of lytic phages based on fundamental biological parameters which rule these interactions. The model is directed to lytic phages only, due to their significance in microbial ecology and in their therapeutic use as antimicrobial agents. The results have shown that the growth rate of the bacterial host plays a critical role in defining the value of the biological parameters of the phage-bacteria population dynamics, mainly the adsorption rate, and consequently in predicting the extent at which phages will grow and control the sensitive bacteria, i.e., the population dynamics of phages and bacteria. Although only the phage adsorption was modelled as a function of the growth rate, the variation of the phage adsorption may also reflect changes in the burst size and latency time that may be a consequence (or not) of that variation. This dependence is able to explain why phages and sensitive bacteria are able to coexist and may be the reason why phages are so successful in controlling bacteria in *in vitro* studies and are not able to achieve such a high performance *in vivo* (where substrate can be limiting). As a consequence, it is essential to understand the influence of environmental conditions (including, but not limited to, substrate availability) on bacterial growth to predict the phage-bacteria behaviour. This dependence will have implications on the use of phages therapeutically and also on their production in bioreactors.

Although the modification of simple models to allow accurate predictions of the *in vivo* interactions of phage and bacteria is complex, the construction of more accurate and precise models will for sure improve our understanding on the role of phages in natural systems and in therapy, diminishing the existing gap between *in vitro* and *in vivo* modelling. Therefore the presented model intends to be a step forward in this domain by introducing variations in the parameters as a function of the bacterial growth rate and also by treating the latent period as a mean value following a normal distribution and not as a single value valid to all the phage-bacteria present, an approximation never included in previous models.

Models explaining the phage-bacteria population dynamics are developed almost exclusively to understand the evolution of these populations in ecological studies and therapy. Nevertheless, in a time where the interest in phages as commercial products is growing exponentially, these models can be of extremely importance in monitoring, control and optimize phage production at large scales.
