# Correction: Population Dynamics of a Salmonella Lytic Phage and Its Host: Implications of the Host Bacterial Growth Rate in Modelling

**DOI:** 10.1371/journal.pone.0136007

**Published:** 2015-08-25

**Authors:** Sílvio B. Santos, Carla Carvalho, Joana Azeredo, Eugénio C. Ferreira

The Fig 1 Figure Legend is incorrect. The authors have provided a corrected version here.

**Fig 1:**
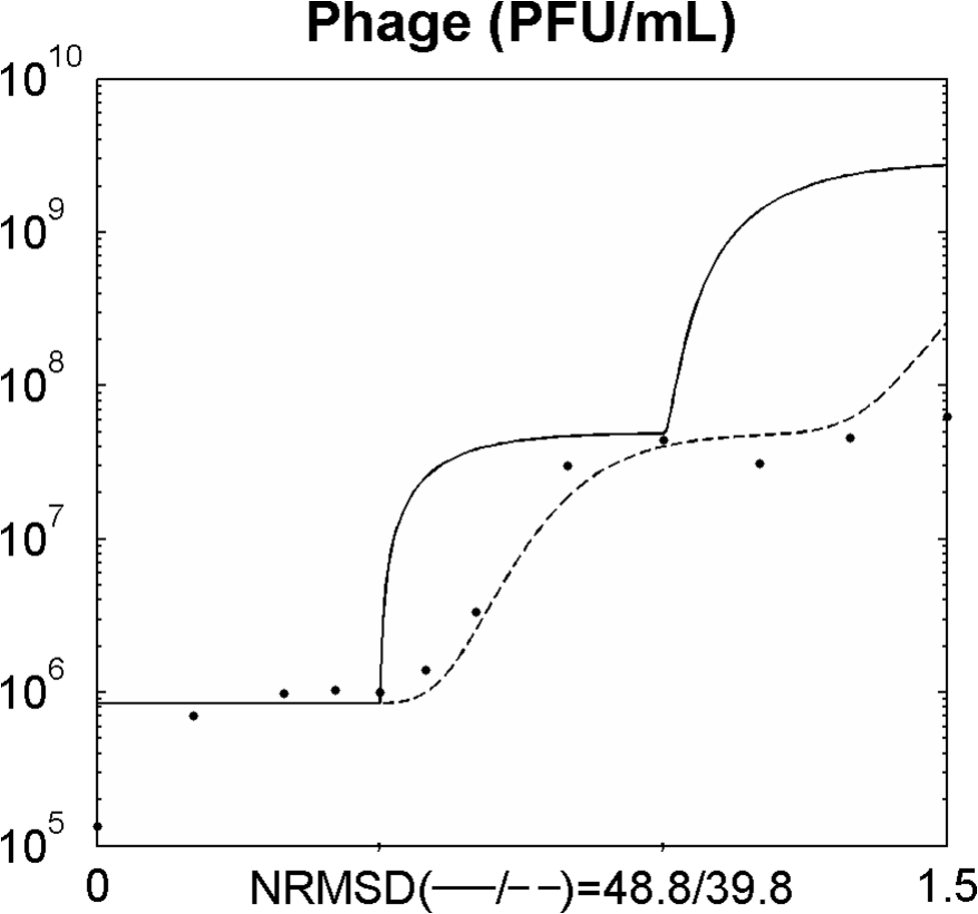
Simulating the one step growth curve using a normal distribution function (equations 6–14). Legend: experimental data; ___ data from the model using the average value of the latent period (equations 6–12);… data from the model using a distribution of values of the latent period (introduction of equations 13 and 14). The x axis represents time in hours.
